# Seismic reflection characteristics and genesis of goafs and underlying coal seams

**DOI:** 10.1038/s41598-026-37861-9

**Published:** 2026-01-30

**Authors:** Rui Shan, Ailan Nie, Xiaoyi Cao, Yunchao Zou, Wei Zhu

**Affiliations:** 1 Coal Technology and Engineering Group Corp., Xi’an Research Institute Co., Ltd., Xi’an, 710077 China; 2 College of Environment and Civil Engineering, Chengdu University of Technology, Chengdu, 610059 China; 3 CCTEG Ecological Environment Technology Co., Ltd., Beijing, 100013 China; 4 College of Geophysics and Petroleum Resources, Yangtze University, Wuhan, 430100 China

**Keywords:** Goaf, Coal seam seismic response, Disturbance effect, Forward modeling, Engineering, Natural hazards, Solid Earth sciences

## Abstract

Coal is an important energy and industrial resource. Coal mining-resulted goafs and subsequently developed caving zones exhibit strong heterogeneity and instability, which can severely restricts the exploration and development of deep coal seams. Focusing on a coal mine in eastern China, this study relied on 2D migrated seismic profiles, and seismic simulating and imaging on a model to systematically investigate the seismic reflection characteristics and its genesis of goafs, caving zones, and their underlying strata. The results indicate that the bottom of goafs presents strong seismic reflections, caving zones generate intense seismic scattering, and reflections from the underlying coal seams exhibit three diagnostic features: namely energy attenuation, phase anomalies, and reduced continuity. Energy attenuation stems from the superimposed effects of strong reflection at the goaf bottom and intense scattering in caving zones. Phase anomalies are dominated by the low-velocity property of goafs and caving zones. Poor continuity is mainly controlled by the scattering in caving zones. The proposed correlation between the attributes of goaf-caving zones and the reflection responses of underlying strata can be used to evaluate the occurrence state of goafs and provide support for the exploration and development of deep coal resources.

## Introduction

Coal serves as both a critical feedstock for the chemical industry and a major energy source, playing an indispensable role in sustaining the operation of human society and driving socioeconomic development. However, underground coal mining inevitably generates large-scale goafs and caving zones (G&CZ), even trigger surface subsidence. These geological hazards pose severe threats to mine safety and human activities in mining areas. Therefore, the reliable characterization of goafs, caving zones, and underlying coal seams is of vital importance for the safe exploitation of deep coal resources.

Three dimensional seismic technologies have pioneered novel avenues for delineating small-scale structures in coal-bearing strata^1,2^. Yuan et al. proposed a time-lapse seismic method for the identification and delineation of G&CZ. By performing seismic forward modeling and imaging on scenarios with and without G&CZ, they verified that these geological features can exert significant perturbations on the reflection signals from underlying formations^3^. Yuan et al. compared post- and pre-stack time migration profiles and concluded that the latter can substantially improve the quality of reflections beneath goafs^4^. Constrained by cost control and other practical factors, 2D seismic exploration is commonly employed as a preliminary survey prior to detailed investigations. It serves as a key tool to assess the detectability of G&CZ, particularly with respect to the clear traceability of their boundaries^5,6^. Under normal circumstances, coal-bearing strata are shallowly buried and associated geological structures are relatively simple, enabling 2D seismic exploration to yield reliable results. However, in the presence of newly formed G&CZ, the fragmented strata can drastically alter the propagation seismic waves. This leads to anomalous features in seismic data, such as energy attenuation, phase disorder, and discontinuous reflectors^7,8^. These anomalies not only obscure structural interfaces but also hinder the accurate identification of minor faults and anomalous boundaries^9,10^. Therefore, in coalfield seismic exploration, accurately identifying the seismic reflection characteristics of G&CZ and their underlying mechanisms is of great practical significance. It contributes to mitigating goaf-related interference, precisely delineating subtle deep-seated coal-seam structures and lithologic variations, and reducing both economic costs and safety risks.

A mining area in eastern China hosts multiple sets of minable coal seams at different depths. Over the past two decades, the exploitation of shallow coal seams has resulted in the formation of numerous G&CZ, accompanied by surface subsidence in some adjacent areas. Prior to the full-scale implementation of seismic exploration, two intersecting seismic lines were deployed in this area. The results indicate that the seismic reflections of the deep coal seams beneath the G&CZ are weak and discontinuous, with significant disturbance effects induced by the G&CZ.

The objective of this paper is to investigate the reflection characteristics of G&CZ under different states. The structure of this paper is organized as follows: Sect. 2 elaborates on the geological overview of a mining area and the geological and petrophysical characteristics of the target coal seams; Sect. 3 introduces the acquisition and processing methods of seismic data; Sect. 4 analyzes the seismic reflection characteristics of G&CZ and their influence laws on underlying coal seams; Sect. 5 employs seismic simulation and imaging to explore the seismic reflection characteristics of G&CZ and their impacts on underlying strata; Sect. 6 presents the main conclusions of this study.

## Basic geology

**Fig. 1:**
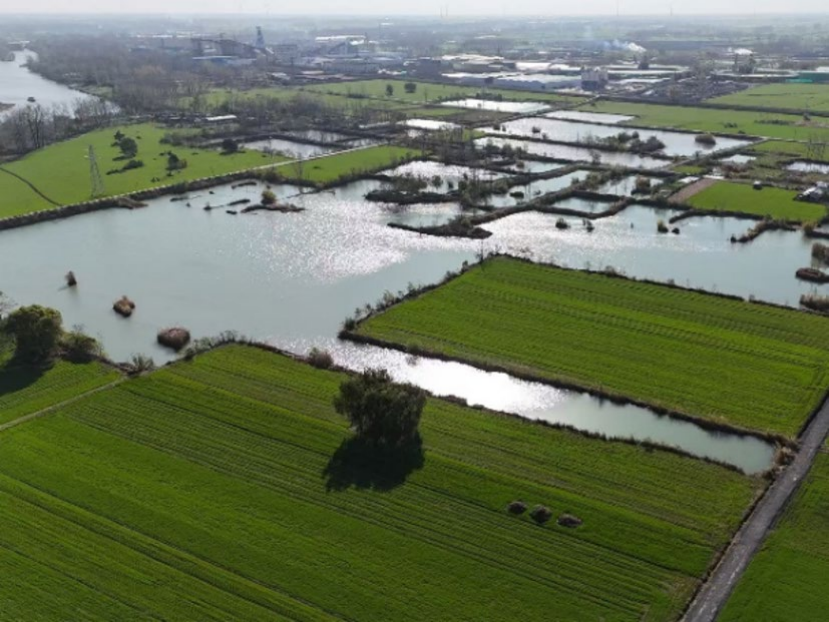
Partial aerial view of the mining area.

The mining area is situated in the Huaibei Plain, featuring a flat terrain and a ground elevation of approximately 25 m. The surface is mantled by unconsolidated deposits, and both surface water and groundwater are relatively abundant; ponds, rivers, and residential settlements are widespread across the area^11^, as shown in Fig. 1.

**Fig. 2:**
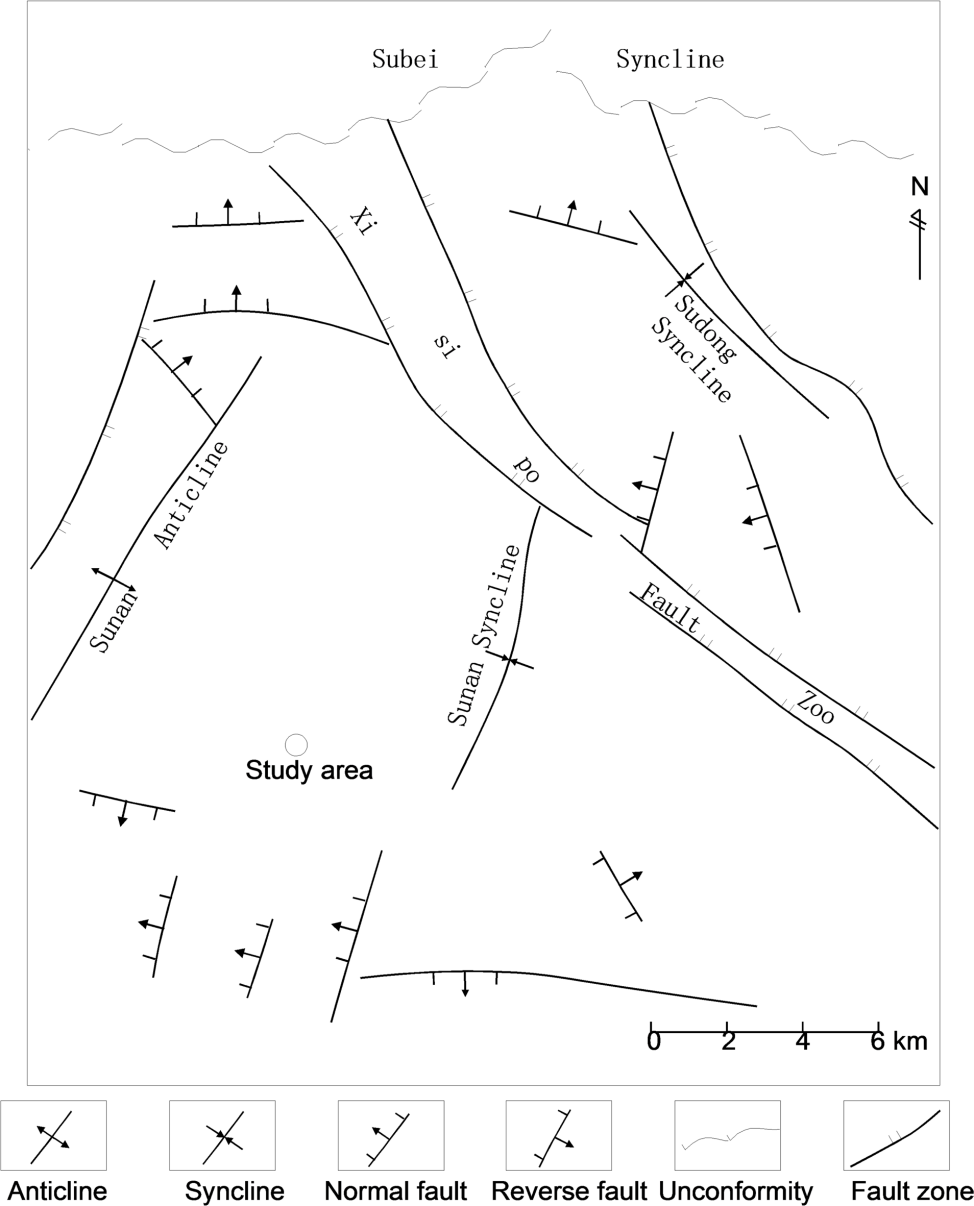
Tectonic outline map of the mining area^11^.

Tectonically, the mining area is located on the southwestern limb of a regional syncline and exhibits a gently plunging structural geometry. Strata strike is predominantly N-S, gradually rotating to a near E-W trend southward; within the strike-transition zone, the fold axis is deflected toward the southwest. The strata dip mainly eastward, progressively turning to a northward to northeastward dip in the southern sector^11^, as shown in Fig. 2.

The stratigraphic succession, from the surface downward, comprises the Quaternary, Neogene, Permian, Carboniferous, and Ordovician systems, with the Permian and Carboniferous forming the principal coal-bearing intervals. The shallow strata, from bottom to top, exhibit the following lithological sequence: multi-cyclic sandstone-clay/sandy clay interbeds, clay or sandy clay strata intercalated with thin-bedded sand bodies, and silt-clay interbeds. The Permian coal-bearing strata are further subdivided, in ascending order, into the Shanxi Formation, Lower Shihezi Formation, and Upper Shihezi Formation. By contrast, the Carboniferous coal seams are generally thin-bedded and characterized by poor stability, rendering most of them either unminable or only marginally minable^11^.

Three primary minable coal seams are developed in the mining area, exhibiting the following vertical distribution characteristics: the upper coal seam lies at a burial depth of 320–420 m with an mean thickness of 2.3 m; its roof is dominated by mudstone, with local siltstone or fine sandstone. The two lower coal seams are buried at a greater depth of 500–900 m, featuring a high thickness variation; their roofs are predominantly mudstone, whereas their floors are sandstone.

**Fig. 3:**
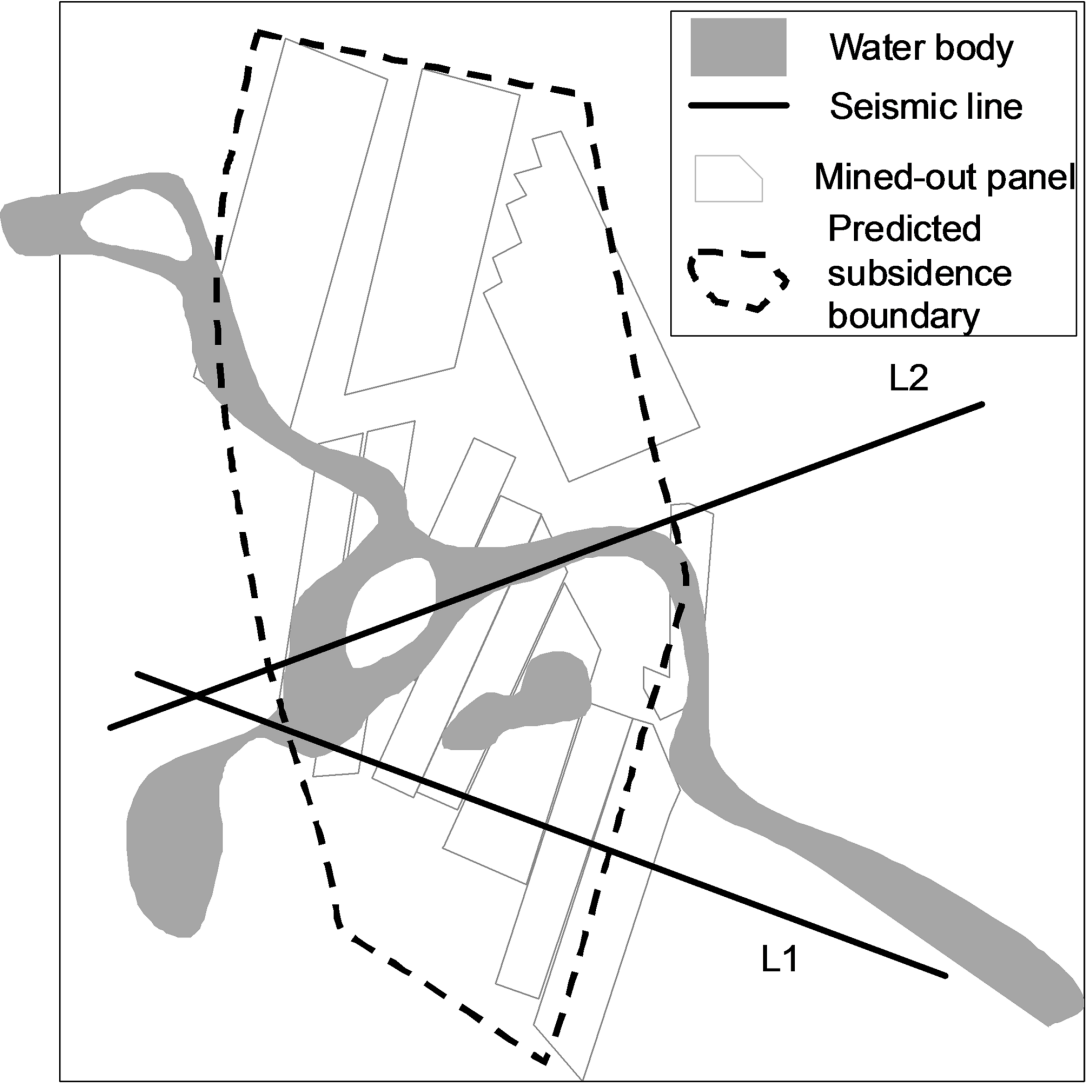
G&CZ distribution map and layout of 2D seismic survey lines.

Following two decades of mining in the upper coal seam, G&CZ are widespread across the area, and extensive surface subsidence has occurred, as shown in Fig. 3.

## Seismic data acquisition and processing

Two 2D seismic lines were deployed in the south-central part of the mining area as shown in Fig. 3. Line L1 strikes northwest-southeast direction, traversing the G&CZ associated with the upper coal seam and extending into the unmined area, with a total length of approximately 2.90 km. Line L2 strikes southwest-northeast direction and is approximately 3.12 km long, with most of the G&CZ located beneath an overlying river channel. Collectively, the two lines cover both the main mined and unmined zones, laying a solid data foundation for characterizing the seismic reflection features of G&CZ.

The key field acquisition parameters are specified as follows: a shot interval of 20 m, a receiver spacing of 10 m, 240 recording channels, a source depth of 10 m, a charge weight of 1.5 kg, a record length of 2.5 s, and a sampling interval of 1 ms.

**Fig. 4:**
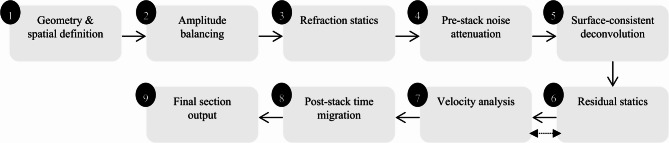
Workflow of seismic data processing.

Although the surface relief is relatively slight, the extensive development of surface water bodies and local ground subsidence may lead to significant variations in the propagation velocity and energy attenuation of near-surface seismic waves. Consequently, static correction and consistency of seismic wavelets are the key aspects requiring focused attention in data processing. The processing workflow is illustrated in Fig. 4.

## Seismic reflection characteristics of G&CZ and their underlying coal seams

### Seismic reflection characteristics in line L1

**Fig. 5:**
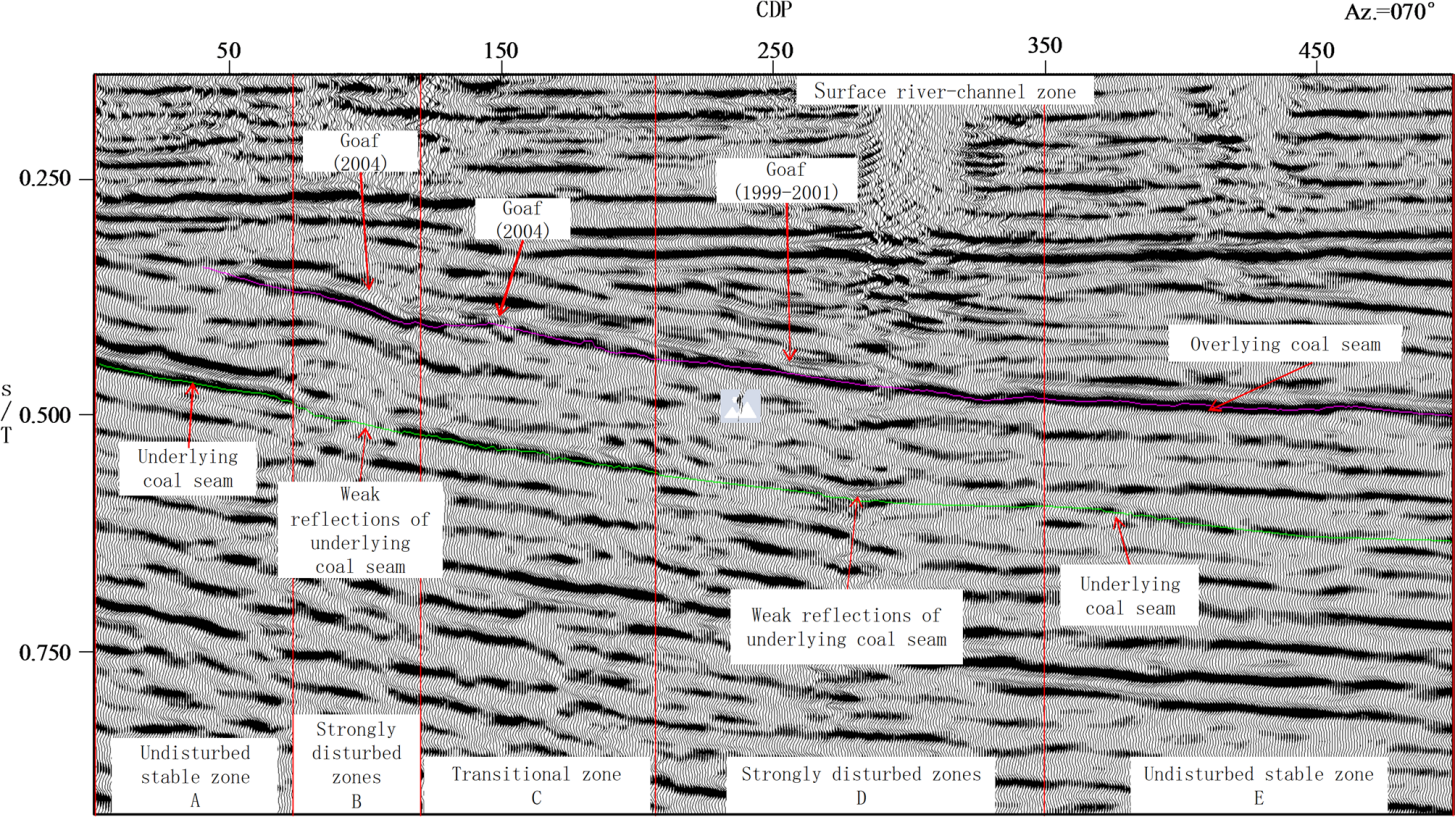
Seismic profile along Line L1.

The migrated seismic profile of Line L1 (segments far from the mining area are not displayed) shows distinct zonal differences between the mined and unmined segments. Based on the continuity of coal-seam reflection events, phase stability, and amplitude distribution, the seismic profile of L1 line was subdivided along the profile into five zones (A to E; Fig. 5).

Zones A and E correspond to comparatively stable segments outside the main goaf influence. Zone A lies outside the mined-out area; the upper seam locally shows a concealed outcrop, whereas reflections from the underlying target seam retain relatively high amplitudes, good continuity, and regular geometries, with no systematic amplitude loss or pronounced phase distortion. In Zone E, reflection events associated with the upper seam remain stable and laterally continuous with comparatively uniform amplitudes and a regular structural pattern; however, the underlying seam exhibits reduced reflection energy, which is a shielding effect caused by strong reflections from the upper seam.

Zones B and D are situated in the central part of the G&CZ and correspond to typical strong disturbed segments. Within Zones B and D, reflections from the upper coal seam exhibit strong amplitude and stable phase. Reflections from the underlying coal seam display phase anomalies and disrupted continuity, indicating a pronounced downward propagation of disturbance effects from the G&CZ.

Zone C lies near the terminal mining line and forms a transition between the central goaf and the surrounding less-disturbed interval. Compared with Zones B and D, the upper-seam reflectors in Zone C show improved continuity, while disturbances affecting the underlying seam are weakened, resulting in a gradual transition from strongly disturbed to relatively stable responses.

### Seismic reflection characteristics in line L2

**Fig. 6:**
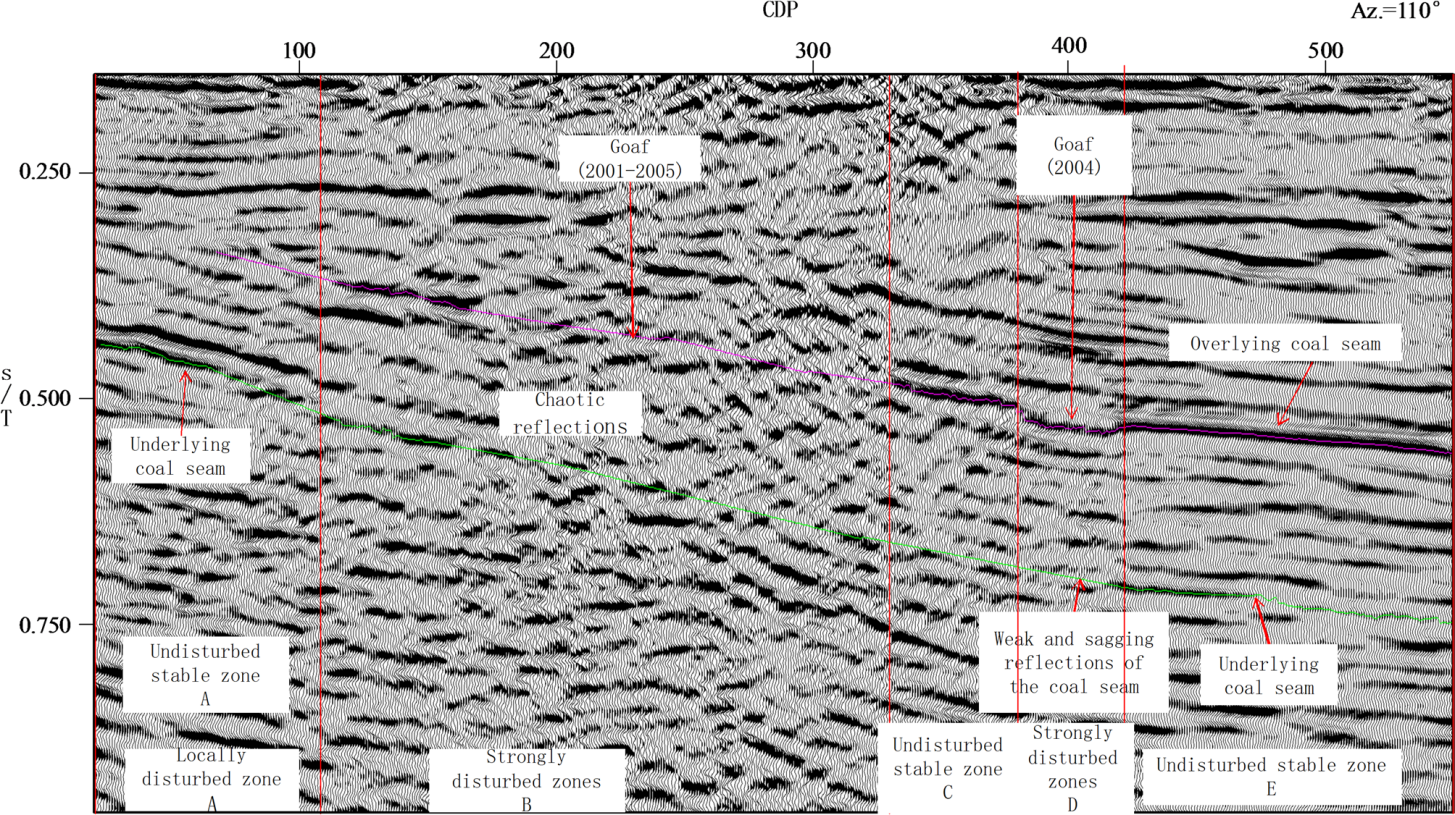
Seismic profile along Line L2.

Applying the same diagnostic criteria (reflector continuity, phase stability, and amplitude characteristics), the seismic profile of L2 was also subdivided into five zones (A to E; Fig. 6).

Zone A is a locally disturbed segment. Although it lies in an unmined area, the profile crosses several transportation and return-air roadways, leading to pronounced amplitude attenuation and reduced continuity in the upper-seam reflectors, whereas most reflections from the underlying seam remain stable, continuous, and geometrically regular.

Zone B is a strongly disturbed segment which should be induced by the G&CZ. The upper-seam reflections are characterized by strong amplitude attenuation and poor continuity, and reflections from the underlying seam show disruption and phase anomalies. This segment coincides with extensive subsidence; progressive overburden caving and abundant residual voids reduce rock-mass integrity and enhance scattering and diffraction, thereby accelerating attenuation and reducing the coherence of deeper reflections. It should be noted that although the overall quality of seismic reflection signals in Zone B is relatively low, the reflection intensity and continuity of the reflection events in the adjacent horizons above the upper coal seam are significantly superior to those of the coal seam.

Zone D is a strongly disturbed segment induced by the G&CZ. In Zone D, the working face is narrower than of Zone B, and upper-seam reflections retain partial continuity, implying a comparatively limited caving extent. Nevertheless, a pronounced floor sag is observed, and reflectors from the underlying seam display downward bending.

Zones C and E are comparatively stable, with laterally continuous upper-seam reflections, relatively uniform amplitudes, and regular structural geometries. Although reflection energy from the underlying coal seam is somewhat reduced due to shielding by strong reflections from the upper seam, these zones remain stable overall.In addition, the underlying coal seam shows lateral thickness variability, which may locally reduce reflector coherence.

### Comparative analysis

The two seismic profiles exhibit consistent patterns in terms of disturbance range, reflection characteristics, and structural responses within G&CZ.

First, both survey lines show clear zonal characteristics. Within G&CZ extent, the reflection events of the upper coal seam generally exhibit energy attenuation, reduced continuity, and local interruption or even disappearance. The underlying coal seam is characterized by significant energy reduction, phase anomalies, and disordered reflection events. This indicates that G&CZ induces intense scattering and attenuation of seismic waves.

Second, the reflection disturbance presents a distinct spatial distribution pattern: the disturbance intensity is the highest in the central part of the goaf, where the disorder of reflection events is most pronounced. When transitioning from the central part of the goaf to its boundaries, the disturbance intensity gradually weakens, and the continuity of the reflection events is progressively restored.

## Seismic wave simulation and imaging of G&CZ

### Construction of G&CZ model

**Fig. 7:**
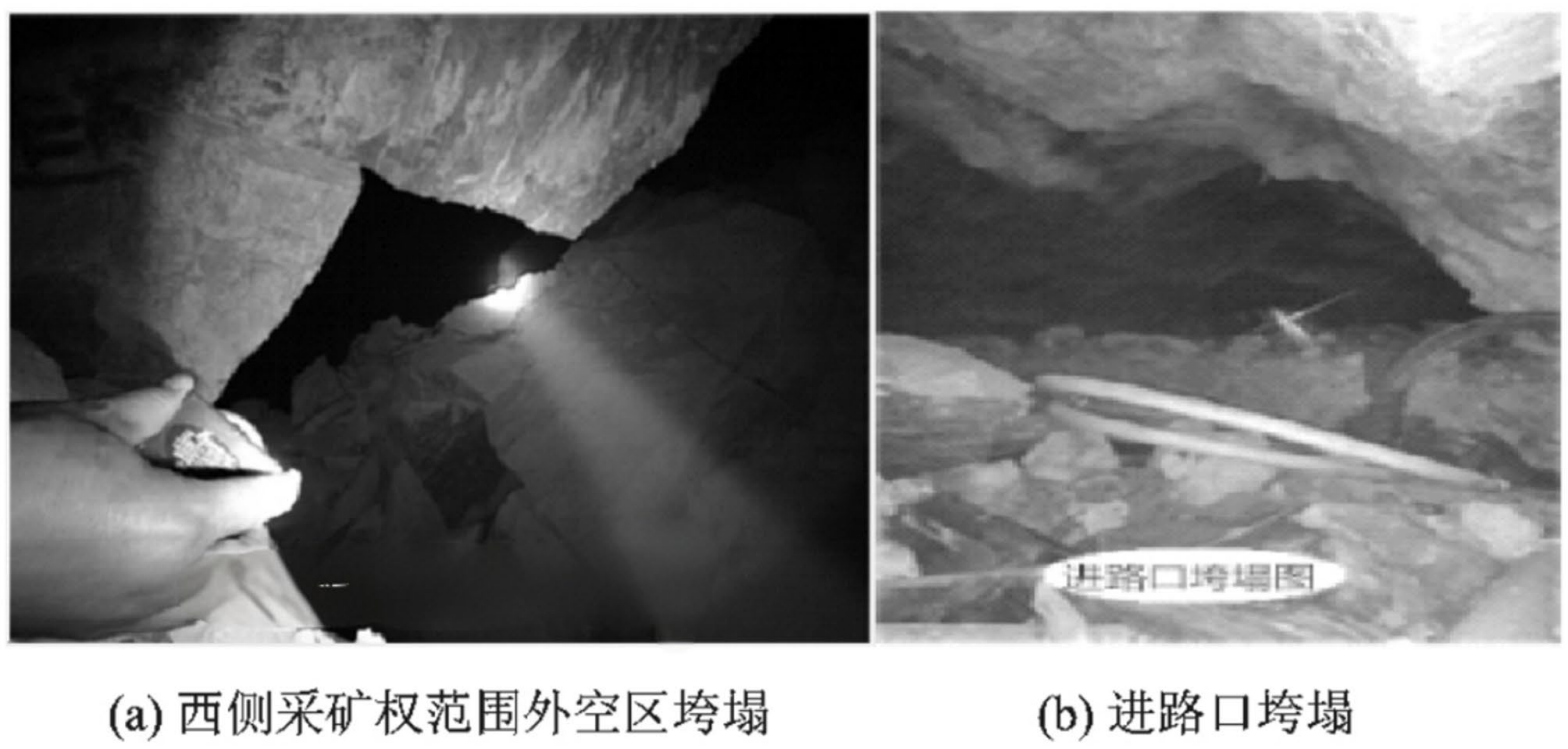
Investigation of the spatial structural evolution laws in large-scale complex goaf area^12^.

**Fig. 8:**
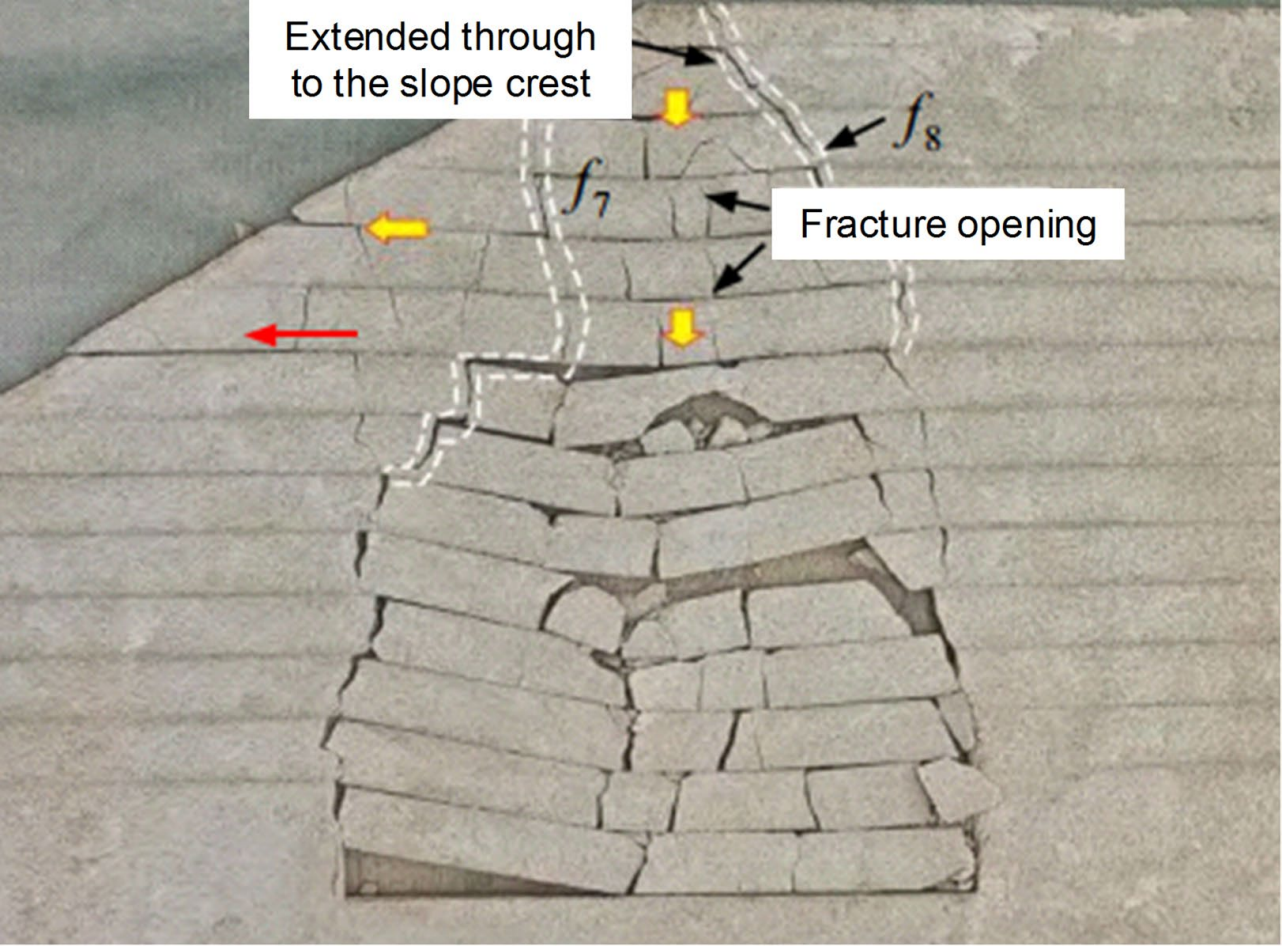
Deformation and structural failure of collapse masses triggered by goaf areas^13^.

The caving zone formed by strata collapse exhibits a complex geological structure. Figure 7 shows a partial photograph of a caving zone, where large rock blocks and voids are clearly visible. Figure 8 presents the results of physical experiments, indicating that the scope of the caving zone may extend to the ground surface, while the distant areas are dominated by fracture development. Combined with the analysis of surface subsidence phenomena, it can be concluded that large-scale caving zones in the mining area has extended to the ground surface.

The strata adjacent to the upper coal seams in the study area are consolidated rock formations, whereas the shallow layer is composed of relatively loose semi-consolidated strata, with significant differences in lithology and mechanical properties between them. Abundant surface water exists in the mining area, making the caving zone prone to groundwater filling. Therefore, there are considerable differences between the forward modeling of seismic waves in G&CZ and that in conventional strata.

The idea of equivalence is adopted to establish the forward model for G&CZ. Within the scope of the caving zone, the P-wave velocity and density of the strata are first reduced, followed by the embedding of discrete blocks with lower P-wave velocity and density. The higher the degree of rock fragmentation, the greater the decreasing of P-wave velocity and density. The P-wave velocity and density of the background strata are determined by comprehensively integrating well logging and seismic data in this area and adjacent. This modeling method is applicable to newly formed caving zones. Over time, the caving zones will be gradually compacted and consolidated, and their impact on seismic wave propagation will consequently weaken.

**Fig. 9:**
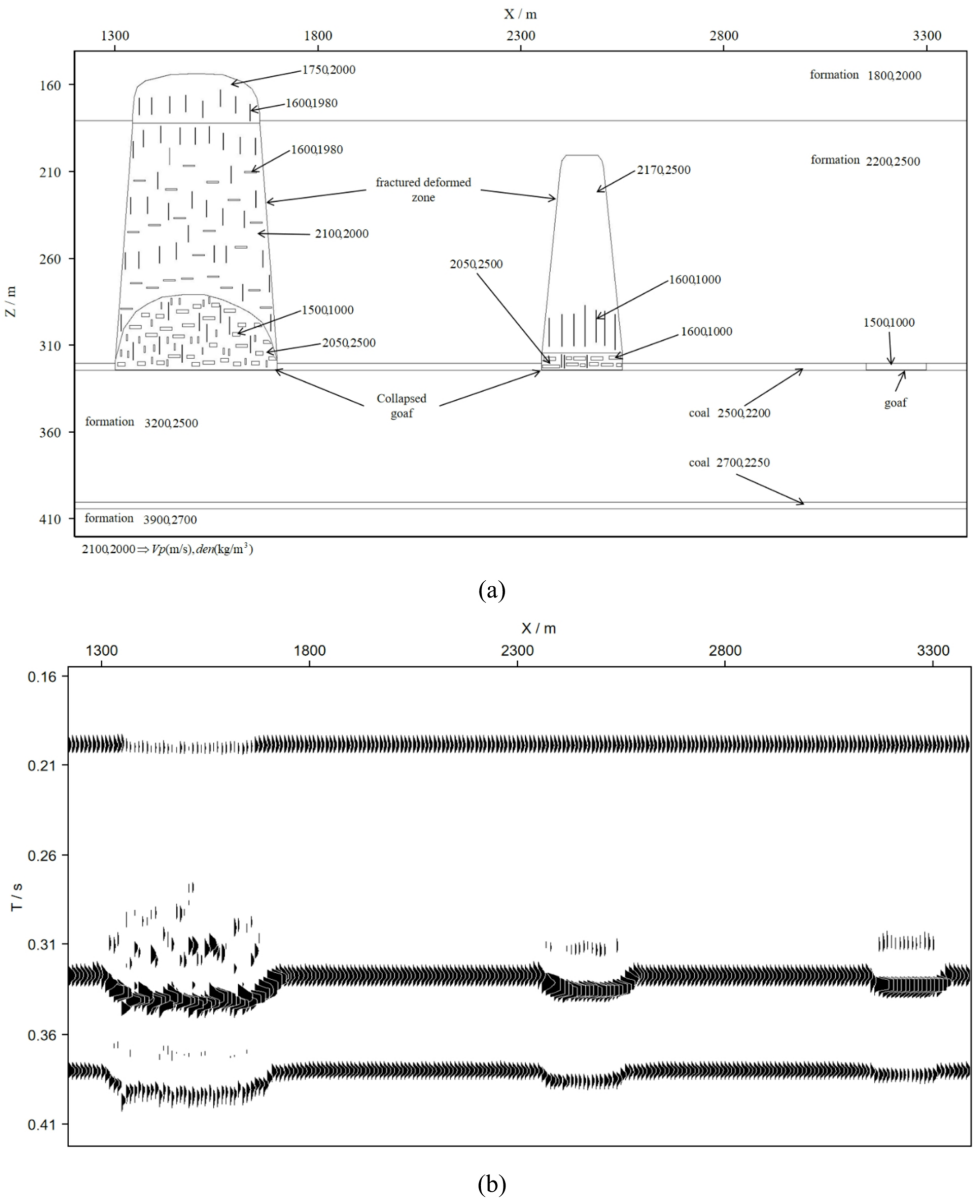
Geological model and migration profile of G&CZ. (**a**) Geological model, (**b**) migration profile.

During the same period, the states of goafs of different scales can exhibit substantial discrepancies. Large-scale goafs collapse to form extensive caving zones, whereas small-scale ones remain structurally intact without collapse. Based on a comprehensive analysis of drilling data and seismic profile interpretation results, an equivalent model including G&CZ is established, as illustrated in Fig. 9a. The background geological model comprises six strata, including two coal seams. Mining activities in the upper coal seam have generated three distinct goafs: the right-hand goaf, with a width of 150 m, remains uncollapsed; the middle goaf, measuring 200 m in width, has collapsed with a relatively limited spatial influence range; and the left-hand goaf, with a width of 300 m, has collapsed and exerts a relatively extensive spatial influence. Both the geometric configuration of the model and the P-wave velocity and density parameters of each geological unit are presented in Fig. 9a.

### Seismic simulation and migration

The seismic wave simulation is implemented using the first-order, velocity-pressure acoustic wave equation and the staggered-grid finite-difference method^14^. The simulation were set as follows: a total of 130 shot points are deployed with a shot interval of 20 m, and the first shot is positioned at *x* = 600 m; the source wavelet is a zero-phase wavelet with a dominant frequency of 40 Hz. For the acquisition geometry, the minimum offset is 20 m, the trace interval is 10 m, and the maximum offset is 810 m; the simulation duration is 1.0 s with a sampling rate of 0.0005 s. The acquisition density of the simulated data is higher than that of field data. Since the simulated data avoided issues including static correction, noise interference and deconvolution, Kirchhoff prestack depth migration is performed on the shot gathers, and the resulting migrated section is presented in Fig. 9b.

### Seismic reflection characteristics of the migration profile

The numerical results reveal the seismic reflection characteristics of G&CZ, as well as the influence on the characteristics of the underlying strata.

The bottom of G&CZ exhibits strong reflection characteristics, whereas the reflected energy of the underlying interfaces undergoes significant attenuation. The caving zones induce scattering effects, which not only generate a chaotic reflected wavefield within the caving zones, but also further attenuate the reflected energy to the underlying interfaces.

The travel time of the reflection events corresponding to both the bottom of G&CZ and the underlying formation exhibits a distinct delay. The reflected wave phases of the formations beneath the caving zone undergo significant distortion, whereas the reflected wave of the interfaces beneath goafs remain essentially consistent.

The continuity of the reflection events corresponding to the formations beneath the caving zone decreases significantly. The reflection events of the interfaces beneath the goaf remain undisturbed and maintain good continuity.

## Conclusion

The seismic reflection anomalies of G&CZ and their underlying coal seams are characterized by three aspects: energy, phase, and continuity of reflection events.

Numerical results indicate that these reflection anomalies are primarily governed by two mechanisms. First is the scattering dissipation effect induced by the complex heterogeneous structure of caving zones; second is the energy shielding effect caused by the strong reflection at the bottom of G&CZ. Among these factors, energy attenuation is attributed to the combined action of scattering dissipation and strong reflection shielding.

Phase distortion and disrupted continuity of reflection events are more sensitive to the development of caving zones. In the case of goafs without caving, reflected waves typically exhibit travel-time delay while maintaining overall waveform consistency. In contrast, in the case of caving zones, reflected waves show significant phase disorder, accompanied by discontinuous distribution of reflection events and a substantial reduction in continuity.

## Data Availability

The datasets generated and/or analyzed during the current study are not publicly available due to confidentiality restrictions.

## References

[1] Lei, C. et al. L. A lithofacies modeling method based on τ-model algorithm and its application. *Reserv. Evaluation Dev.* **13**(2), 206–214 (2023).

[2] Liu, Y. M. et al. Application of artificial intelligence lithofacies prediction in carbonate rock petrophysical modeling. *Oil Geophys. Prospect.* **58**(S1), 125–131 (2023).

[3] Yuan, Y., Gao, Y., Bai, L. & Liu, Z. Prestack Kirchhoff time migration of 3D coal seismic data from mining zones. *Geophys. Prospect.* **59**, 455–463 (2011).

[4] Yuan, H., Liu, J. & Yuan, Y. Using 4-D seismic data for detecting gob areas of coal mines: A case study from the Zhangji coal mine. *IEEE Trans. Geosci. Remote Sens.* **60**, 5917810 (2022).

[5] Jia, N. Study on seismic wave propagation and permeability characteristics of strata damaged by mining [PhD thesis]. Liaoning Technical University, (2024).

[6] Wang, A., Fu, C., Li, G., Wang, H. F. & Chen, T. J. Comparative study on locating Goaf boundaries by seismic attributes. *Coal Technol.* **42**(10), 31–35 (2023).

[7] Cao, X. S. Seismic response characteristics of Goaf and its lower coal seam. *Coal Chem. Ind.* **46**(1), 35–38 (2023).

[8] Cao, X. S., Shen, Y. X., Guo, P. P. & Zhang, Z. Application of 3D seismic in exploration of lower coal seam under double-layer Goaf. *Coal Chem. Ind.* **46**(2), 38–41 (2023).

[9] Liang, J. M. Study on surface movement and deformation law of goaf under fault influence [PhD thesis]. China University of Mining and Technology (2024).

[10] Zhang, Z. Z. Stability analysis of coal pillars in strip goaf and study on surface deformation law in Daizhuang Coal Mine [PhD thesis]. China Coal Research Institute (2024).

[11] Guo, Y. H. Study on surface prevention and control of coal mining subsidence range in Huaibei Plain mining area [PhD thesis]. Anhui Jianzhu University (2025).

[12] Deng, H. W., Wang, Y. & Xu, Y. H. Study on the Spatial structural evolution law of large complex Goaf. *J. Disaster Prev. Mitigation Eng.* **38**(3), 401–408 (2018).

[13] Sun, S. W. et al. Mechanism of slope failure induced by subsidence of underground Goaf in coal mines. *Chin. J. Rock Mechan. Eng.* **44**(6), 1405–1419 (2025).

[14] Li, X. Study on forward modeling of seismic wavefield and full waveform inversion method in undulating surface [PhD thesis]. China University of Petroleum (Beijing) (2022).

